# Cost-utility analysis of a dance intervention for adolescent girls with internalizing problems

**DOI:** 10.1186/1478-7547-11-4

**Published:** 2013-02-20

**Authors:** Anna Philipsson, Anna Duberg, Margareta Möller, Lars Hagberg

**Affiliations:** 1 Department of Community Medicine and Public health, Örebro County Council, P.O. Box 1613, SE-70116, Örebro, Sweden; 2 Centre for Health Care Sciences, Örebro County Council, Örebro, Sweden; 3 School of Health and Medical Sciences, Örebro University, Örebro, Sweden

**Keywords:** Internalizing problems, Adolescent girls, Physical activity, Dance, Cost-utility analysis

## Abstract

**Background:**

The increasing prevalence of psychological health problems among adolescent girls is alarming. Knowledge of beneficial effects of physical activity on psychological health is widespread. Dance is a popular form of exercise that could be a protective factor in preventing and treating symptoms of depression. The aim of this study was to assess the cost-effectiveness of a dance intervention in addition to usual school health services for adolescent girls with internalizing problems, compared with usual school health services alone.

**Methods:**

A cost-utility analysis from a societal perspective based on a randomized controlled intervention trial was performed. The setting was a city in central Sweden with a population of 130 000. A total of 112 adolescent girls, 13–18 years old, with internalizing problems participated in the study. They were randomly assigned to intervention (n = 59) or control (n = 53) group. The intervention comprised dance twice weekly during eight months in addition to usual school health services. Costs for the stakeholder of the intervention, treatment effect and healthcare costs were considered. Gained quality-adjusted life-years (QALYs) were used to measure the effects. Quality of life was measured with the Health Utility Index Mark 3. Cost-effectiveness ratios were based on the changes in QALYs and net costs for the intervention group compared with the control group. Likelihood of cost-effectiveness was calculated.

**Results:**

At 20 months, quality of life had increased by 0.08 units more in the intervention group than in the control group (*P* = .04), translating to 0.10 gained QALYs. The incremental cost-effectiveness ratio was USD $3,830 per QALY and the likelihood of cost-effectiveness was 95%.

**Conclusions:**

Intervention with dance twice weekly in addition to usual school health services may be considered cost-effective compared with usual school health services alone, for adolescent girls with internalizing problems.

**Trial registration:**

Name of the trial registry: “Influencing Adolescent Girls’ With Creative Dance Twice Weekly”

Trial registration number: NCT01523561

## Background

A trend of increasing mental health problems and psychosomatic symptoms among children and adolescents has been shown in recent years [1,2]. Several indicators demonstrate that internalizing problems, such as anxiety and depression, are particularly common among young women, reported by as many as 20 percent of adolescent girls, [2,3]. The number of adolescent girls (10–19 years old), diagnosed in institutional care with depression and anxiety is alarming; it has more than tripled in Sweden during the past ten years [4]. Internalizing problems may impose high societal costs, disability and decreased quality of life for individuals [5-7]. Research on interventions targeting adolescent girls is scarce and there is a great need for effective interventions [8]. Furthermore, the society has the right to demand that interventions offered are based on scientific evidence and that they are cost-effective [9-11].

The School Health Services comprise a physician and a school nurse and its aim is to “monitor the students’ development, to protect and improve their mental and physical health, and to try to instill healthy living habits in them” during the whole school-age period from 6 to 19 years of age [12]. Visits to the School Health Services by the students, especially girls, are common and research shows that most of the visits to the school nurse are caused by headache, depression, back or neck problems, or by students who just “want to talk” [13]. The activities of the School Health Services are stated in the Education Act and are also included in The principles of health care of the National Board of Health and Welfare [14,15].

Studies show that physical exercise in general can be an active strategy to prevent and treat depression and anxiety and to promote positive thoughts and feelings [16-19]. Dance is a popular form of exercise for girls [20,21]. Research has also shown that dance could be a protective factor in preventing and treating symptoms of depression [22,23]. No economic evaluation has been found of dance as an intervention to prevent or treat internalizing problems.

The aim of this study was to assess the cost-effectiveness of a dance intervention in addition to usual school health services for adolescent girls with internalizing problems, compared with usual school health services alone.

## Methods

### Intervention trial

A complete description of the intervention trial has been published elsewhere [24].

#### Study design

**Figure 1:**
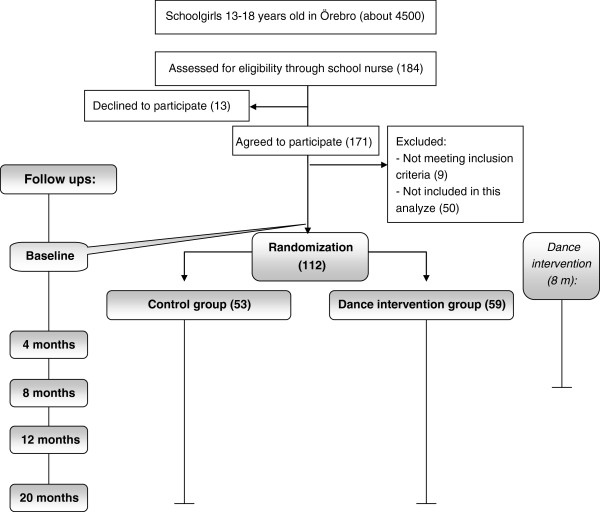
**Flowchart.** Participants responded to a baseline questionnaire at the start of the study. Randomization was carried out, and the 8-month dance intervention was initiated. Follow-ups for both groups were at 4, 8, 12, and 20 months after baseline.

The study was a prospective, randomized and controlled intervention trial with an intervention group who received dance classes twice weekly during 8 months, and a control group who received standard prevention and care. Participants were followed up five times during the study period (Figure 1). The Regional Ethical Review Board in Uppsala, Sweden has approved the trial (DNR 2008/134).

#### Study population

Girls 13 to 18 years old with internalizing problems, i.e. often experiencing feelings of stress, who visited the school nurse frequently or who had recurrent psychosomatic symptoms, were enrolled. Persistent feelings of tiredness, being worried, in low spirit or low mood were also inclusion criteria. Exclusion criteria were severe hearing impairment, mental retardation, difficulties in speaking or writing in the Swedish language, and advice against inclusion by the Children and Adolescent Psychiatry or a psychologist [24].

The dance intervention was conducted twice in order to offer more girls the intervention and to overcome the limit in resources, such as the number of dance instructors and studio availability. Hence, participants were enrolled and randomized on two different occasions. However, the analyses in this article include only the girls who participated in the intervention in the first year and the new intake girls who participated in the intervention during the second year. The girls who were randomized to the control group in the first year and crossed over to the intervention group in the second year, were excluded from the analyses. The study took place in a city in central Sweden with 130 000 citizens [24].

A total of 112 participants were included in the analyses. Baseline characteristics are described in Table 1. An external statistician randomly allocated the participants to the intervention group (n = 59) or the control group (n = 53). The reason for allocating more participants to the intervention group was that we wanted to make sure that there would be a sufficient number of girls to fill up the dance groups. There was no blinding. All self-reported data were entered into the computer by a research assistant [24].

**Table 1:** Baseline characteristics

**Variable**	**Intervention group** (**n** = **59**)	**Control group** (**n** = **53**)
	**N** (%)	**N** (%)
Age ^*a*^ 13-14 years	8 (13)	13 (25)
Age 15–16 years	27 (46)	23 (43)
Age 17–18 years	24 (41)	17 (32)
Born in Sweden	55 (93)	49 (93)
Lives with both parents	24 (41)	30 (57)
Rates their health as poor or very poor	8 (14)	3 (6)
Experiences feeling of stress frequently	41 (69)	28 (53)
Participated in dancing before start of study	33 (56)	36 (68)

^a^ Mean for both intervention group and control group: 16 years.

#### Dance intervention

The intervention comprised dance classes twice weekly during 8 months. It was pronounced from the start of the intervention that the focus was on joy and not on performance. The dance classes consisted of different dance themes and each session contained warm-up, creative group practice, dance routine and stretch. Each session was ended with relaxation [24].

#### Usual school health services

Usual school health services comprised conventional prevention and care provided by the school nurse according to the guidelines for the School Health Services in the county of Örebro [25]. The objective of these guidelines is to clarify the routines and the guiding principles to ensure equal treatment of the students. Another objective is to define evidence-based methods for the School Health Services according to the Health and Medical Service Act [15]. Both the intervention and control group had access to this conventional prevention and care.

### Economic evaluation

The economic evaluation was performed as a cost-utility analysis from a societal perspective, using individual data [26]. In the analysis costs for the stakeholder of the intervention, health effect and savings in health care use were considered (Table 2). Gained quality-adjusted life-years (QALYs) were used to measure the effects. Cost-effectiveness ratios were based on the changes in quality of life (QOL) and net costs for the intervention group compared with those for the control group. QOL was measured with the Health Utility Index Mark 3 (HUI3) [27]. The time period measured was 20 months. Health effects and savings beyond 12 months were discounted by 3%.

**Table 2:** **Factor**, **variable and method for the economic evaluation**

**Factor**	**Variable**	**Method**
Intervention costs	Intervention costs for the stakeholder	Individual costs were calculated based on the number of participants in the intervention group, and estimated fractions of costs for dance teacher, rent, equipment and overhead [28,29].
Health care savings	Health care savings for stakeholder	Savings for health care were estimated based on self-reported data from the last term before baseline and 20 months use after the start of the intervention. Number of visits was compared with the baseline value at all follow-ups. Costs for each visit were calculated based on estimated costs for salary, etc [30,31]. and total number of visits to the school nurse.
Health effect	QOL	HUI3

#### Description of costs

Cost estimates were derived through contacts with personnel in the project and in the head office of the School Health Services [28-31]. The cost estimates considered in this analysis were: (1) the cost of the dance classes; (2) the cost of using selected healthcare resources, i.e. visits to the school nurses (self-reported values). Number of visits was compared with the baseline value at all follow-ups. All cost estimates, however, included overhead costs and were converted from Swedish krona (SEK) to USD using an approximated exchange rate of 1 SEK = 0.15 USD. The prices are valid for 2011.

#### Description of health effect

The Health Utilities Index Mark 3 (HUI3) [27,32,33] was used to measure and ascribe values to the participants’ health states. The HUI3 is a pre-scored health status instrument, originally developed in Canada, that asks the patient questions about his or her overall health status and health-related quality of life The HUI3 assesses eight preference-based attributes of function (health status) that are believed to contribute to quality of life: Ambulation, Dexterity, Speech, Vision, Hearing, Cognition, Emotion, and Pain. Each attribute has five or six levels of ability/disability. These levels are ordered from normal function (not impaired, or a ranking of 1) to severely impaired (a ranking of 5 or 6, depending on the attribute). All attributes are structurally independent, which means that all combinations of levels in the system are possible, allowing descriptions of 972 000 health states [27,32,33]. In the present study, the attributes emotion, cognition and pain were determined to be the most suitable for the study subject [27]. The time frame captured was ‘current health’ (no specified period). Single questions were used for each attribute. The original version of the HUI3 questionnaire was developed in English [27,32,33]. For the present study, the original Canadian questionnaire was translated to Swedish by a professional translator, after which another translator translated it back to English. The original and the back-translated English versions were compared and checked for discrepancies, after which the Swedish questionnaire was revised.

Gained QALYs were calculated from the difference in QOL between the intervention and control groups at all follow-ups. The overall utility score from the HUI3 was used as the quality adjustment factor for calculating QALYs gained. Differences were assumed to develop linearly between follow-ups.

#### Cost-effectiveness

Cost-effectiveness ratio (ICER) is expressed as Costs – savings / QALY = ICER.

Cost-effectiveness is often calculated using the mean difference in costs and effects, and is based on comparison between two or more possible treatment options. Using mean values for cost-effectiveness ratios is associated with uncertainty. In the analysis, this uncertainty is handled with the Net Monetary Benefit (NMB) method [34,35]. This method is based on health effects (QALYs) given a value, on individual level, of that amount of money decision makers are willing to pay for a gained QALY. When all data are expressed in money, it is possible to calculate the likelihood that an intervention is cost-effective in relation to a competing intervention.

### Statistical methods

Data were analyzed according to the intention-to-treat principle, meaning that all study participants were analyzed in the study group that they were randomized to. Analyses of effect size within each group were performed using paired-samples t tests. Differences between groups were analyzed with independent-samples t tests. Individual values were used for savings in healthcare, gained QALY and costs in intervention and control groups. Significance level was set at *P < .05*.

A scatter plot of 5 000 bootstrapped incremental cost-effectiveness ratios was created by repeatedly drawing a random sample with replacement, using parameters estimated from the randomized controlled intervention trial. This produced estimates of the probability that the intervention was cost-effective using several thresholds of willingness to pay for a QALY. Minitab 15.0 was used for this process. Results are presented in a cost-effectiveness acceptability curve [36].

If data were missing the last observation carried forward approach was used [37-39]. This implies that the last value observed is imputed.

### Sensitivity analyses

We conducted sensitivity analyses. Cost-effectiveness was also calculated without savings, with 50% higher costs and with 50% reduced effect on QALYs. For NMB, we repeated the estimation of net monetary benefit with two different amounts for how much a gained QALY can cost.

## Results

112 subjects were included in the study and 93 (83%) of them completed the 20-month follow-up.

### Intervention costs

The mean cost for one girl participating in one dance session was USD $25 (Table 3). Based on that value the mean cost for the whole dance intervention was USD $670 per participant. As the control group didn’t receive any dance intervention there were no such costs for them.

**Table 3:** **Costs per each dance session per participant** (**USD**)

**Type of cost for the intervention**	**Cost**
Dance teacher	14.6 ^a^
Rental for dance studio	8.1
Equipment (music and continuation courses)	0.2
Further education for the dance teachers	0.2
Overhead costs 13%	1.9
Total costs per each dance session per participant	25

^a^ Including social fees.

### Healthcare costs

The cost for each visit to the school nurse was estimated at $58. The mean number of visits to the school nurse was estimated to 20,00 in the intervention group and 27,83 in the control group based on baseline values. The mean number of visits to the school nurse decreased by 10,75 (−53,75%) in the intervention group, compared with baseline values. In the control group, the mean number of visits decreased by 6,89 (−24,82%).

### Health effect

**Figure 2:**
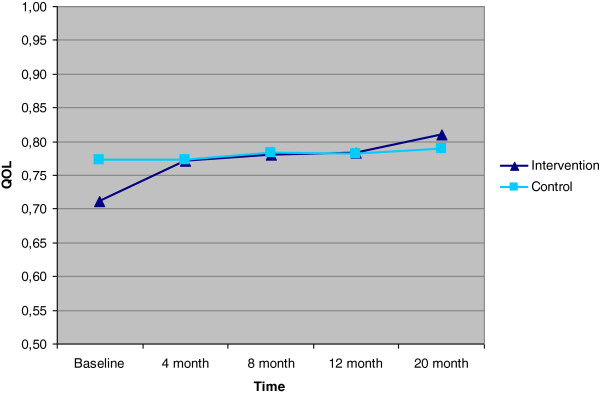
QOL, based on HUI3 for intervention and control groups.

**Figure 3:**
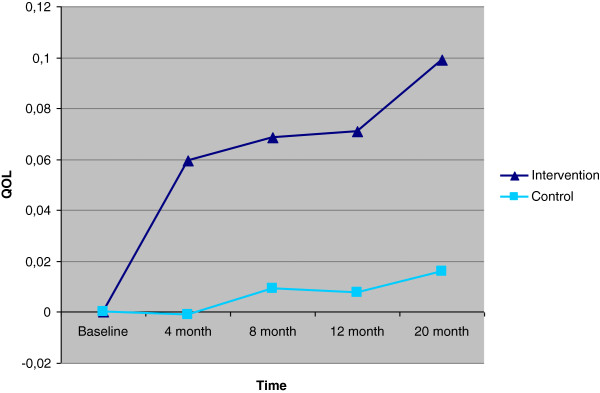
Change in QOL from baseline, based on HUI3 for intervention and control groups.

After 20 months, the participants in the intervention group had increased their QOL by 0.08 units more than those in the control group (*P* = .04) (Table 4). The increase in the intervention group was greater than that in the control group at all follow-ups (Figures 2 and 3). Based on this data, gained QALY at 20 months was 0.10 (*P* = .03).

**Table 4:** **Treatment effect in QOL based on the HUI3 for intervention** (**I**) **and control** (**C**) **groups**

	**Baseline**	**20 months**, **change from baseline**
Intervention (I)	0.71 (0.66 – 0.77)	0.10 (0.05 – 0.15)
Control (C)	0.77 (0.73 – 0.82)	0.02 (−0.05 – 0.08)
Difference I - C	−0.06 (−0.01 – 0.13)	0.08 (−0.16 – -0.01)

### Cost-effectiveness

The net costs per participant in the intervention group were $383 at 20 months. Costs per gained QALY were $3,830 after 20 months (Table 5).

**Table 5:** **Results of cost**-**utility analysis for a 20**-**month period**

**Variable**	**After 20 months**
Gained QALY	0.10 (0.01 – 0.18)
Intervention costs	670 (570–770)
Savings in health care (compared to control group)	287 (−735 – 1310)
Net costs	383
Costs per gained QALY	3830
Costs per gained QALY (healthcare savings excluded)	6700
Costs per 50% of gained QALY	7660
Cost per gained QALY with 50% increased intervention costs	7180

**Figure 4:**
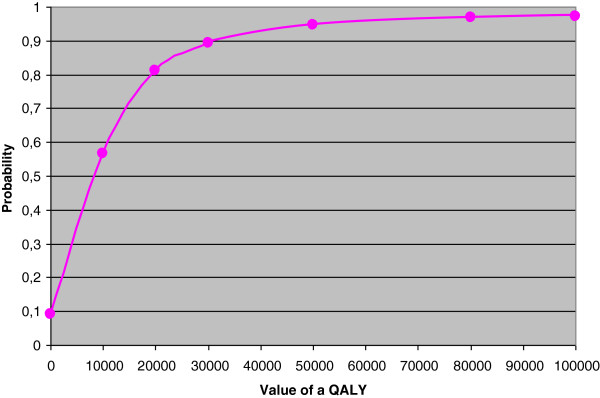
**Probability of cost-effectiveness.** Probability of cost-effectiveness using the HUI3 presented in a cost-effectiveness acceptability curve with 0, 10 000, 20 000, 30 000, 50 000, 80 000, and 100,000 USD as value of a QALY.

The probability of cost-effectiveness when stakeholders are willing to pay 50,000 USD for a QALY [40] is 95% (Figure 4).

## Discussion

### Main results

When considering the first 20 months, the ICER is $3,830 per QALY. The dance intervention was shown to increase the QOL, which translated into a QALY gain of 0.10 after 20 months. In addition to an increase in QOL, sustained new healthy habits for this target group might prevent future psychiatric illness [41]. The aim of this analysis was not to evaluate which elements of the intervention that was important for the effect.

There is no official level of willingness to pay for a gained QALY in the United States, but $50,000 and $100,000 are often used [40]. In Sweden, an unofficial threshold of $75,000 has been used to guide decisions about subsidized medicine [42]. The ICER of $3,830 per QALY in this study is consequently well below that threshold. These results indicate that the dance intervention was valuable and an efficient use of healthcare resources in relation to what Western countries are willing to pay for a gained QALY.

Healthcare consumption, i.e. number of visits to the school nurse, was an uncertain factor. A test of the factor showed that there was a considerable gap between the number of visits reported by the girls and the number of visits found in the medical records. The school nurses expressed difficulties with the charting of social and mental health issues, due to lack of time, tradition, structure of the journal, and ethical considerations [31,43]. The self-reported visits were based on a retrospective open question which can be unvalid. Therefore, the ICER was calculated without these, giving an ICER of $6,700. The sensitivity analysis also showed that the ICER was doubled ($7,660) when using 50% of gained QALY and $7,180 when costs were assumed to be 50% higher than calculated, still well below the threshold value.

In this analysis, only savings for the School Health Services were considered. The larger increase in QOL in the intervention group may also have led to savings for other institutions such as primary care, youth centers, and the total Welfare Services in school. Since research has shown associations between internalizing problems in adolescence and in adulthood, it can also be assumed that the larger increase in QOL after the dance intervention could lead to further savings [44-46]. However, the present study was too small to be able to support this notion.

The girls included in the study were obviously exposed to outside influences in addition to the intervention. For example, periods of examinations in school may have increased feelings of stress and thereby affected the girls’ general well-being. Since the intensity of school work varies during the school year, the 12-month follow-up can be considered the most important one.

### Strengths and weaknesses

The economic evaluation was performed as a cost-utility analysis from a societal perspective, based on a randomized controlled trial. This method is adopted to find out how you can optimally allocate limited resources and is suitable when the aim is to compare different kinds of interventions. It is also a preferred approach for stakeholders compared with other common models [26,47].

The HUI3 is a well recognized instrument, published in detail [27,32,33,48,49], and providing a good estimate of utility values in a community-dwelling, relatively healthy, population. It has been developed by using preferences from a random sample of respondents 16 years of age and older and has also been used to measure health status in several studies on children [48]. However, a weakness is that the validity and reliability of the Swedish version of the instrument have not been tested. We did not agree with the translation in the official Swedish version of the word ‘unhappy’. In the official version, ‘nedstämd’ (similar to lightly depressed) was used, but we chose to use what we believe is a more common expression among youth, ‘olycklig’ (similar to not happy or not glad).

A strength of this analysis is its long-term perspective. There is a great need for studies that establish the sustainability of interventions, to evaluate whether the effects can remain for a long period after the end of the intervention. In this case the 20-month follow-up was approximately a year after the last session of the intervention.

Extrapolation of the trial data was necessary. The last observation carried forward approach was used to handle missing data. This approach has been criticised in recent years for weaknesses in validity and estimations of the result [37,39,50] . Other methods were considered but the last observation carried forward approach was chosen since both groups in the study were feeling better after 20 months. Since the participants in the study in general showed an increase in QOL, it can be assumed that the results are more likely to be underestimated than overestimated, which some of the critics claim often is the case.

It cannot be ruled out that differences in QOL at baseline, despite not being significant, may have had an impact on the cost-effectiveness ratio. A lower value at baseline means a higher possible increase. Moreover, it cannot be ruled out that a control group with exactly the same QOL at baseline would have increased their QOL more than the control group in our study. Hence, some caution in interpreting the results is recommended.

Differences in gains of QOL between the intervention and the control group were 0.06 units at the end of the intervention and 0.08 one year later (follow-up at 20 months after start). Hence, a sustainable effect of the intervention seems to be possible, which would in that case give a lower ICER. However, we have not found any research which indicates for how long time the effect may remain. We don’t think it is likely that the effect will end the day after the last follow-up. On the other hand, assumptions of very long-lasting effects may be an overestimation.

In the way the intervention was organized in this study, there were no costs for the participants. The dance hall was close enough to the participants’ schools to not require any travelling costs. The girls in the intervention reported high enjoyment of the dance sessions [24]. This indicates that there might not have been any sacrifice, such as loss of enjoyment compared to an alternative activity, to participate in the dance sessions.

### Results in relation to previous research

No economic analyses were found of dance as an intervention to prevent or treat internalizing problems. This analysis therefore seems to be filling a gap of knowledge.

The scarceness of evidence-based interventions for adolescent girls with internalizing problems may be due to the lack of studies as well as organizational complexity, with many institutions involved in prevention and care. However, the patient group is in great need of effective interventions.

### Further research required

The present study evaluated the cost-effectiveness of an intervention with dance for adolescent girls with internalizing problems. Intervention studies and economic analyses of interventions for this target group is an unexplored research area, in great need of further studies. Specifically for this study, there is also a need to find out what the most important components of the dance interventions were.

The results of this research can support comparisons with other approaches to prevent or treat mental problems as well as with other modalities. The research also highlights the need for long-term research to establish the sustainability of this type of interventions.

The present study also revealed that there is a lack of research on the school health services in Sweden.

## Conclusions

The present study shows that, for adolescent girls with internalizing problems, it can be cost-effective to complement the school health services with a dance intervention. The dance intervention was shown to increase QOL, and the increase remained 20 months after start. Costs per QALY were rather low and if you assume that the effect will remain over time, the ICER will be even lower.

## Abbreviations

QALY: Quality-adjusted life-years;QOL: Quality of life;HUI3: Health Utility Index Mark 3;ICER: Incremental cost-effectiveness ratio

## Competing interests

The authors declare that they have no competing interest.

## Authors’ contributions

AD, LH and MM drafted the concept and design of the study, and conducted the data collection. AD and MM designed the intervention. AP performed the health economic analysis. AP wrote the manuscript and AD assisted. LH provided health economic expertise, assisted in the economic evaluations, and supervised the writing, analysis and revision. All authors read and approved the final manuscript.
